# Detection and Isolation of Swine Influenza A Virus in Spiked Oral Fluid and Samples from Individually Housed, Experimentally Infected Pigs: Potential Role of Porcine Oral Fluid in Active Influenza A Virus Surveillance in Swine

**DOI:** 10.1371/journal.pone.0139586

**Published:** 2015-10-02

**Authors:** Inge Decorte, Mieke Steensels, Bénédicte Lambrecht, Ann Brigitte Cay, Nick De Regge

**Affiliations:** 1 Operational Direction Viral Diseases, Enzootic and (re)emerging diseases, CODA-CERVA, Ukkel, Belgium; 2 Operational Direction Viral Diseases, Avian virology and immunology, CODA-CERVA, Ukkel, Belgium; University of Liverpool, UNITED KINGDOM

## Abstract

**Background:**

The lack of seasonality of swine influenza A virus (swIAV) in combination with the capacity of swine to harbor a large number of co-circulating IAV lineages, resulting in the risk for the emergence of influenza viruses with pandemic potential, stress the importance of swIAV surveillance. To date, active surveillance of swIAV worldwide is barely done because of the short detection period in nasal swab samples. Therefore, more sensitive diagnostic methods to monitor circulating virus strains are requisite.

**Methods:**

qRT-PCR and virus isolations were performed on oral fluid and nasal swabs collected from individually housed pigs that were infected sequentially with H1N1 and H3N2 swIAV strains. The same methods were also applied to oral fluid samples spiked with H1N1 to study the influence of conservation time and temperature on swIAV infectivity and detectability in porcine oral fluid.

**Results:**

All swIAV infected animals were found qRT-PCR positive in both nasal swabs and oral fluid. However, swIAV could be detected for a longer period in oral fluid than in nasal swabs. Despite the high detectability of swIAV in oral fluid, virus isolation from oral fluid collected from infected pigs was rare. These results are supported by laboratory studies showing that the PCR detectability of swIAV remains unaltered during a 24 h incubation period in oral fluid, while swIAV infectivity drops dramatically immediately upon contact with oral fluid (3 log titer reduction) and gets lost after 24 h conservation in oral fluid at ambient temperature.

**Conclusions:**

Our data indicate that porcine oral fluid has the potential to replace nasal swabs for molecular diagnostic purposes. The difficulty to isolate swIAV from oral fluid could pose a drawback for its use in active surveillance programs.

## Introduction

H1N1, H1N2 and H3N2 IAV subtypes have become enzootic in swine in many countries worldwide, causing explosive outbreaks of respiratory disease characterized by pyrexia, anorexia, lethargy, dry coughing, sneezing, rhinorrhea and often growth retardation. The clinical signs associated with IAV infection of pigs cause substantial economic losses due to the increased time needed to attain slaughter weight [1], making the disease a key concern for the swine industry.

Pigs are important hosts in the ecology of IAV since they are susceptible to infection with influenza viruses from both avian and human origin [2, 3]. Co-circulation of different influenza strains in the same production batch can lead to reassortment of the genome segments, increasing the diversity of the circulating influenza strains [4–11]. These reassortant viruses can demonstrate phenotypically particular characteristics, which might result in facilitated inter-species transmission [12]. Since 2009, multiple independent introductions of the pandemic H1N1/09 virus (pH1N1) into swine have led to several reassortant H1N1, H1N2 and H3N2 viruses in swine worldwide [6, 13–19]. Some of these reassortant viruses became established and continued to circulate within the affected production sytems [7, 14]. Although recent evidence shows that IAV transmission from humans to pigs occurs more frequent than swine-to-human IAV transmission [3], the recent human cases caused by novel reassortants of pH1N1 and swine H3N2 viruses are of particular concern [20–23]. The continuous circulation of pH1N1 in swine increases the chance of further reassortment what could result in a novel reassortant virus with the potential to cause infection and efficient transmission among humans [7].

Contrary to IAV infections in humans, IAV infections in swine occur throughout the year [24, 25]. This lack of seasonality of swIAV infection in combination with the capacity of swine to harbor a large number of co-circulating IAV lineages from different hosts stress the importance of IAV surveillance in pigs and the early detection of newly emerging swIAV strains. Therefore, systematic surveillance of pig populations and a thorough analysis of IAV in swine is recommended [3, 6]. Active surveillance of influenza in swine populations is however barely done because of the technical and economic challenges of testing a statistically relevant number of pigs [24]. Yet, information obtained from surveillance activities could play a pivotal role, not only for a better understanding of endemic and emerging influenza virus ecology but also to make influenza isolates available for research, for updating diagnostic assays and for vaccine development [26]. Consequently, IAV surveillance in swine requires the monitoring of circulating virus strains using sensitive and reliable diagnostic methods. RT-PCR (reverse transcription real-time PCR) and virus isolation (VI) on nasal swab samples are currently the primary methods used to detect swIAV. Nasal virus shedding in swine is most likely to be found during the febrile period of illness (mostly between 1 to 4–5 dpi) and this narrow time frame poses one of the most important challenges in swine influenza diagnostics [27]. Only recently it was shown that influenza virus could be detected in pen-based oral fluid samples from experimentally and naturally infected pigs [28]. That study showed that pen-based oral fluid samples were RT-PCR positive for swIAV till 6 days post infection (dpi), but unfortunately, no samples at later time points were collected. Another study on pen-based oral fluid samples by Goodell et al. [29] showed that the probability of detecting SIV in oral fluids and nasal swabs by rRT-PCR was equivalent till 6 dpi and then higher in pen-based oral fluid samples till 16 dpi. Since no information is available from individually housed animals, it was our objective to compare the detection of swIAV strains in oral fluid and nasal swabs collected during experimental infection of individually penned animals by qRT-PCR and virus isolation on embryonated chicken eggs and Madin-Darby canine kidney (MDCK) cells. Further laboratory studies were also conducted to study the influence of conservation time and temperature on swIAV infectivity and PCR detectability in porcine oral fluid.

## Material and Method

### Virus titration

Sw/Gent/28/10 (H1N1) (4^th^ and 5^th^ passage) and Sw/Gent/172/08 (H3N2) (4^th^ passage), two strains representative of swIAV in Belgium, were propagated in the allantoic cavity of 10-day-old embryonated chicken eggs (ECE). The virus stocks were titrated in ECE. Briefly, eggs were inoculated with 100 μL of 10-fold virus stock dilutions made in PBS supplemented with antibiotics (10^7^ U/L penicillin, 10 g/L streptomicin, 0.25 g/L gentamicin). For each dilution, 5 eggs were inoculated. After 7 days of incubation at 37°C, allantoic fluid was tested for hemagglutinating activity with 0.5% chicken erythrocytes. Titers were calculated following the method of Reed and Muench. The H1N1 stocks contained 1 x 10^8.8^ EID_50_ / mL (4^th^ passage) and 1 x 10^9.7^ EID_50_ / mL (5^th^ passage). The H3N2 stock contained 1 x 10^9.5^ EID_50_ / mL.

### Effect of oral fluid on virus infectivity

To determine the potential effect of conservation time and temperature on infectivity of swIAV present in oral fluid, oral fluid collected by ropes from swIAV negative pigs (determined by qRT-PCR, ELISA and hemagglutination inhibition (HI) assays) via the same method as described further was spiked (9:1) with the H1N1 virus stock (5^th^ passage) and conserved either at 4°C or at room temperature (22°C ± 2°C). At 0, 0.5, 2, 6 and 24 h after spiking, aliquots of 2 mL were taken and 222μL PBS supplemented with antibiotics (10^8^ U/L penicillin, 100 g/L streptomicin, 2.5 g/L gentamicin) was added (9:1) and incubated 1 h at room temperature. Afterwards 10-fold dilutions were made in PBS supplemented with antibiotics (10^7^ U/L penicillin, 10 g/L streptomicin, 0.25 g/L gentamicin), and per dilution, 5 eggs were inoculated with 100μl each. The titration was further performed as described above. As a control, a homologous experiment was performed in which the H1N1 virus stock was spiked in PBS instead of oral fluid.

### Effect of oral fluid on virus detectability via qRT-PCR

To study the potential effect of oral fluid on swIAV detectability via quantitative reverse transcription real-time PCR (qRT-PCR), the H1N1 stock (5^th^ passage) was spiked in oral fluid collected from swIAV negative pigs to a final concentration of either 1 x 10^8.7^ EID_50_ / mL or 1 x 10^4.7^ EID_50_ / mL. The spiked samples were conserved either at 4°C or at room temperature (22°C ± 2°C) and at 0, 0.5, 2, 6 and 24 h after spiking, aliquots were collected, stored at -80°C and tested via qRT-PCR as described further. As a control, a homologous experiment was performed in which the H1N1 virus stock was spiked in PBS instead of oral fluid. Three independent replicates of this experiment were performed.

### Animals, inoculation and sample collection

Sixteen Belgian Landrace piglets were purchased at the age of 8 weeks from a commercial swine herd known to be free of PRRS virus (TaqMan NA and EU PRRSV Reagents, Life Technologies) and negative for PRRSV-specific antibodies (PRRS X3 Ab Test, Idexx). Upon entry in the air-filtered level–2 biosecurity facilities (CODA-CERVA Machelen), piglets were randomly assigned to the control and infection group and housed individually on slatted floors. Prior to the start of the experiment, pigs were confirmed seronegative to swIAV as determined by a commercial ELISA (Influenza A Ab Test, Idexx). After one week of acclimatization, ten piglets were manually restrained and 0.5 mL of 10^8.8^ EID_50_/mL of the H1N1 strain (sw/Gent/28/10, 4^th^ passage) was administered in each nostril by aerosol inoculation with a small plastic nebulizer (length: 4 cm; spray opening: 1 mm). The remaining six pigs were left uninoculated and served as negative control animals. Three weeks later, the inoculated group was infected with the H3N2 strain (sw/Gent/172/08) by aerosol inoculation of 1 mL of 10^9.5^ EID_50_/mL (0.5 mL per nostril). To analyze virus excretion, nasal swabs were collected from all pigs five days before inoculation, as well as on 0, 1, 2, 3, 5, 7, 10, 14 and 21 days post infection (dpi) with the H1N1 strain and at 0, 1, 2, 3 and 5 dpi with the H3N2 strain. Nasal swab samples were suspended in 1 mL modified Eagle medium (MEM) (Life technologies), mixed vigorously at 4°C for 1h and stored at -80°C until further use. Oral fluid samples were collected at the same time points following the method described by Prickett et al. [30] with the exception that our samples were collected with 1 meter long polyester ropes (diameter: 16 mm; colour: white with blue pattern; Barrois-Cebef, Brussels) and not with cotton ropes. Oral fluid samples were immediately chilled on ice, centrifuged at 1800 x *g* for 10 minutes and stored as aliquots at -80°C until use. At 5 dpi with the H3N2 strain all animals were euthanized by electrocution followed by exsanguination.

This study was performed in accordance with EU and Belgian regulations on animal welfare in experimentation. The protocol was approved by the joined Ethical and Biosecurity committee of the Belgian Institute of Public Health and CODA-CERVA (procedure agreement no. 120112–01).

### Quantitative reverse transcription real-time PCR

For the detection of swIAV, qRT-PCR was performed using an M gene-targeted commercial RT-PCR kit, following the kit protocol. Briefly, RNA from nasal swabs was extracted using the MagMAX Pathogen RNA/DNA kit (Life Technologies) according to the manufacturer’s protocol. RNA from oral fluid was extracted as previously described [31]. Following extraction, the RNA was amplified with the Vetmax Gold swIAV detection kit (Life Technologies) in a 25 μL reaction mixture using 8 μL of extracted RNA. All PCRs were run on a LightCycler 480 Real-time PCR system (Roche). In each run, a 10-fold dilution series of the positive control (10.000 copies/μl) present in the PCR kit and negative control samples were tested with the unknowns. Samples with a Ct < 38 were considered positive. For the oral fluid samples collected during the in vivo experiment, the obtained Ct values were converted into copy numbers/mL using a linear regression that was fitted to the Ct values obtained for the dilution series of the positive control and taking the dilution factors introduced during sample preparation and RNA extraction into account.

### Virus isolation

Nasal swab samples and oral fluid samples with a Ct value < 30 were analyzed by virus isolation on ECE and MDCK cells. Standard methods were used to isolate swIAV on ECE [32]. Briefly, after centrifugation of nasal swabs and oral fluids, PBS supplemented with antibiotics (10^8^ U/L penicillin, 100 g/L streptomicin, 2.5 g/L gentamicin) was added to the samples (1:9; 100 μL supplemented PBS + 900 μL oral fluid) and incubated 1h at room temperature. 200 μL of the supernatant was inoculated in quadruplicate into the amniotic cavity of 9 to 10-day-old ECE and incubated at 37°C for 5 days. Eggs were monitored daily for mortality and at day 5 virus growth was detected by using a hemagglutination assay on the allantoic fluids. HA-positives were subsequently subtyped by HI assays.

For isolation of swIAV in MDCK cells from nasal swabs, samples were diluted 3:1 in MEM (Sigma-Aldrich) supplemented with antibiotics and antimycotics (200 IE/mL penicillin, 100 μg/mL gentamicin, 0.5 μg/mL amphotericin B) (= complete MEM) (180 μL sample + 60 μL complete MEM). Oral fluid was 1:3 diluted in complete MEM (60 μL oral fluid + 180 μL complete MEM). Diluted samples were held for 60 min at 21°C before inoculating the cells. Confluent monolayers of MDCK cells were prepared in 24-well plates, washed three times with complete MEM and each well was inoculated with 200 μL of the diluted samples. After a 2h absorption period at 37°C in a 5% CO_2_ humidified incubator, cells were washed with complete MEM and 1 mL of cell culture maintenance medium (complete MEM supplemented with 0.5 μg/mL TPCK-treated trypsin (Sigma)) was added. The cell cultures were observed daily for the appearance of cytopathic effect (CPE) for 7 days. After 7 days, the supernatant was removed and all samples were tested by qRT-PCR.

### Statistical analyses

2-sided Fisher’s exact tests were used to evaluate differences in swIAV detection ratios in nasal swabs and oral fluid by qRT-PCR at each time point. Independent-samples t-tests were conducted to compare i) the number of oral fluid samples collected from swIAV-infected and mock infected piglets and ii) the log copy numbers in nasal swabs and oral fluids at different time points post infection. Data were analyzed using SPSS Statistics V22.0 (IBM) software and P values < 0.05 were considered to be significant.

## Results

### Sample collection

**Fig 1:**
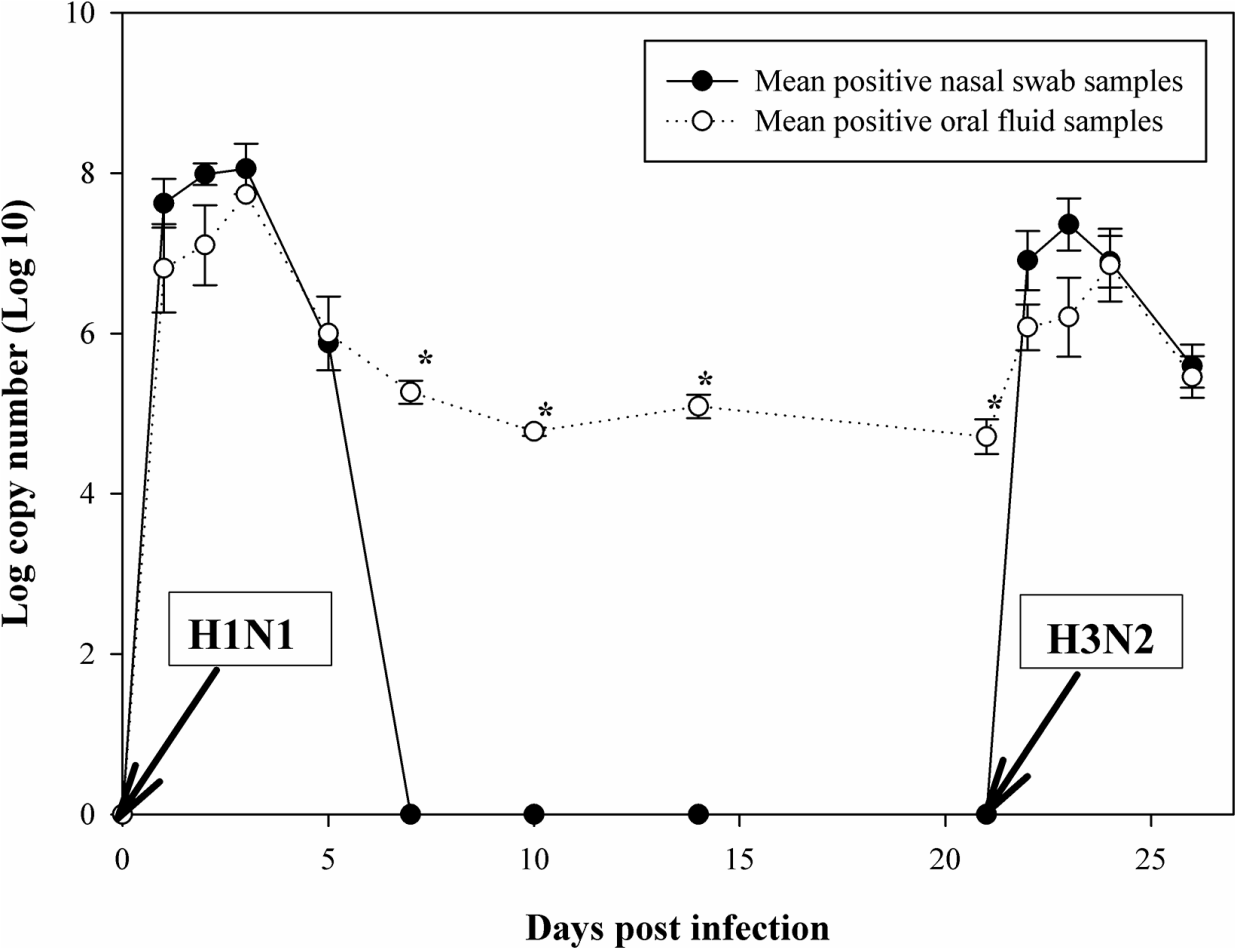
Mean log copy number/mL detected in nasal swab samples and oral fluid samples of pigs sequentially infected with swine influenza A virus strains sw/Gent/28/10 (H1N1) and sw/Gent/172/08 (H3N2). Mean log copy number/mL of viral RNA detected by real-time PCR in nasal swab samples (black circles) and oral fluid samples (white circles) collected from individually housed pigs sequentially infected with the sw/Gent/28/10 (H1N1) strain at day 0 and the sw/Gent/172/08 (H3N2) strain at day 21. Data show the mean (± standard error of the mean) of positive samples. Asterisk denotes a significant difference, as determined by the independent samples T-test (*p* < 0.05).

During the study period, nasal swabs (n = 224) were collected from all pigs at each indicated time point. Despite our efforts to collect oral fluid samples from all piglets at each time point, only 108 oral fluid samples were obtained (Table 1) since piglets were repeatedly not interested to chew on the presented rope. The mean number of samples collected per piglet did not significantly differ between swIAV-infected and mock infected piglets (mean_infected, mock infected_ = 6.7, 6.8; P = 0.95), indicating that swIAV infection did not influence the biting behavior of the pigs. Nevertheless, individual oral fluid samples from infected piglets were most difficult to obtain at 3 dpi with H1N1 (1 sample from 10 pigs) and H3N2 (3 samples from 10 pigs) strains (Table 2), respectively. This time point corresponded to the peaks of virus excretion (Fig 1).

**Table 1:** Successful oral fluid collections by ropes from individually housed piglets aged between 8 and 13 weeks.

swIAV-infected pigs										Mock-infected control pigs					
1	2	3	4	5	6	7	8	9	10	1	2	3	4	5	6
6*	7	7	4	7	10	6	1	13	6	11	1	1	13	4	11

*The results represent the number of successful collections on 14 different sampling days (1 collection attempt/day)

**Table 2:** Detectability of swine influenza A virus RNA by qRT-PCR and virus isolation in nasal swab samples (A) and oral fluid samples (B) of pigs sequentially infected with swine influenza A strains sw/Gent/28/10 (H1N1) at day 0 and sw/Gent/172/08 (H3N2) at day 21.

Virus strain	H1N1 (dpi)	0	1	2	3	5	7	10	14	21	22	23	24	26
	H3N2 (dpi)									0	1	2	3	5
**A**	**Pig 1**	-	+	+	+^†^ ^,^ ^1^ ^,^ ^2^	-	-	-	-	-	+^†^ ^,^ ^2^	+^†^ ^,^ ^2^	+^†^ ^,^ ^2^	-
	**Pig 2**	-	+	+	+	-	-	-	-	-	+	+	+	-
	**Pig 3**	-	+	+^†^ ^,^ ^2^	+^†^ ^,^ ^1^ ^,^ ^2^	-	-	-	-	-	-	+	+	-
	**Pig 4**	-	+^†^ ^,^ ^1^ ^,^ ^2^	+^†^ ^,^ ^2^	+^†,^ ^1^ ^,^ ^2^	+	-	-	-	-	+	+^†^ ^,^ ^2^	+	+
	**Pig 5**	-	+	+	+	-	-	-	-	-	-	-	-	-
	**Pig 6**	-	+^†^ ^,^ ^2^	+^†^ ^,^ ^2^	+^†^ ^,^ ^1^ ^,^ ^2^	-	-	-	-	-	+	+	+	-
	**Pig 7**	-	+^†^ ^,^ ^2^	+	+	-	-	-	-	-	+	+^†^ ^,^ ^2^	+	+
	**Pig 8**	-	+	+^†^ ^,^ ^1^ ^,^ ^2^	+^†^ ^,^ ^1^ ^,^ ^2^	-	-	-	-	-	+	+	+	-
	**Pig 9**	-	+^†^ ^,^ ^1^ ^,^ ^2^	+^†^ ^,^ ^2^	+^†^ ^,^ ^2^	-	-	-	-	-	+	-	-	-
	**Pig 10**	-	+	+	+	-	-	-	-	-	+	+	+	-
**Positive detections**		**0/10**	**10/10**	**10/10**	**10/10**	**1/10**	**0/10**	**0/10**	**0/10**	**0/10**	**8/10**	**8/10**	**8/10**	**2/10**
**B**	**Pig 1**	ns	+^†,^ ^2^	+^†^	ns	+	+	ns	+	+	ns	ns	ns	ns
	**Pig 2**	ns	+	+	ns	-	ns	-	ns	-	+^†^	+^†^	ns	ns
	**Pig 3**	-	+^†^	+^†^	ns	ns	+	ns	+	+	ns	ns	ns	ns
	**Pig 4**	ns	ns	ns	ns	ns	ns	ns	ns	ns	+	+^†^	+	+
	**Pig 5**	ns	ns	+	ns	+	ns	+	-	-	-	+	ns	ns
	**Pig 6**	-	+^†^ ^,^ ^2^	+^†^	ns	+^†^	+	ns	ns	-	+	+	ns	+
	**Pig 7**	-	ns	ns	ns	ns	ns	-	-	-	+	ns	ns	+
	**Pig 8**	ns	ns	ns	ns	ns	ns	ns	-	ns	ns	ns	ns	ns
	**Pig 9**	-	+^†^ ^,^ ^2^	+	+^†^	+	+	+	+	-	+	+	+	-
	**Pig 10**	ns	ns	ns	ns	ns	+	ns	-	-	ns	+	+	+
**Positive detections**		**0/4**	**5/5**	**6/6**	**1/1**	**4/5**	**5/5**	**2/4**	**3/7**	**2/8**	**5/6**	**6/6**	**3/3**	**4/5**
**Fisher’s exact test (P)**		Ϯ	Ϯ	Ϯ	Ϯ	0.02^‡^	0.00^‡^	0.07	0.05	0.18	1.00	0.50	1.00	0.09

ns: No sample available; +: PCR pos; -: PCR neg

^†^: Samples with a Ct value < 30 submitted for virus isolation on embryonated chicken eggs (EVE) and MDCK cells

‡: Significant difference between swIAV detection rates in nasal swab samples and oral fluid at that particular time point (p<0,05)

Ϯ: No measure of association is computed because both variables are constant

^1^: Samples analyzed by virus isolation on ECE with positive result

^2^: Samples analyzed by virus isolation on MDCK with positive result

### Detection of swine influenza A virus by quantitative reverse transcription real-time PCR in samples from experimentally infected piglets

Prior to the inoculation, all oral fluids and nasal swabs tested negative by qRT-PCR and all samples from the negative control animals remained negative throughout the trial. Between 1 and 3 dpi with the H1N1 strain, all nasal swab samples (30/30) and all oral fluid samples (12/12) collected were swIAV positive by qRT-PCR (Table 2). At 5 dpi, only 1/10 (10%) nasal swabs was found positive while 4/5 (80%) oral fluid samples were still positive. From 7–21 dpi none of the 40 nasal swab samples were positive while 5/5 (100%), 2/4 (50%), 3/7 (42.9%) and 2/8 (25%) of the oral fluid samples were positive at 7, 10, 14 and 21 dpi, respectively. After inoculation with the H3N2 strain, 21 days post H1N1 infection, viral RNA was detected in 24/30 (80%) of the nasal swabs collected between 1–3 dpi and in 2/10 (20%) nasal swabs collected at 5 dpi. Detectability in oral fluid went from 83.3% (5/6) at 1 dpi with the H3N2 strain to 100% (9/9) at 2 and 3 dpi and to 80% (4/5) at 5 dpi, respectively. Fisher’s exact tests showed that significantly more oral fluid samples were H1N1 positive by qRT-PCR than nasal swab samples at 5 and 7 dpi. At 10 and 14 dpi, the detection rate between nasal swabs and oral fluid was not significantly different. However, P values of 0.07 and 0.05 at those respective time points suggest that swIAV might be longer detectable in oral fluid than in nasal swabs.

Not significantly different amounts of swIAV RNA were found in nasal swabs and oral fluid during the first five dpi for both strains, while significant higher copy numbers were found in oral fluid from 7 dpi onwards (*p* < 0.05) (Fig 1; S1 Table).

### Detection of swine influenza A virus by virus isolation in samples from experimentally infected piglets

swIAV could be isolated from 8/15 (53.3%) and 15/15 (100%) nasal swabs collected after the H1N1 infection on ECE and MDCK cells, respectively (Table 3). In contrast, the H1N1 strain could not be isolated from oral fluid samples on eggs (0/9) and only from 3/9 (33.3%) oral fluid samples on MDCK cells. All three positive oral fluid samples were collected at 1 dpi. When virus isolation was attempted from nasal swabs and oral fluid collected from H3N2 infected pigs, swIAV was isolated from all nasal swabs with a Ct value <30 on MDCK cells (5/5) but not on ECE (0/5). All isolations from oral fluid (3) remained negative, both on MDCK cells and on ECE.

**Table 3:** Comparison of swine influenza A virus isolation from oral fluid samples and nasal swab samples collected from pigs sequentially infected with (A) swine influenza A virus strains sw/Gent/28/10 (H1N1) at day 0 and (B) sw/Gent/172/08 (H3N2) at day 21 in embryonated chicken eggs (ECE) and in Madin-Darby canine kidney (MDCK) cell culture.

A. H1N1	MDCK					
	Oral fluid			Nasal swab		
ECE	Positive	Negative	Total	Positive	Negative	Total
Positive	0	0	0	8	0	8
Negative	3	6	9	7	0	7
Total	3	6	9	15	0	15
B. H3N2	MDCK					
	Oral fluid			Nasal swab		
ECE	Positive	Negative	Total	Positive	Negative	Total
Positive	0	0	0	0	0	0
Negative	0	3	3	5	0	5
Total	0	3	3	5	0	5

Overall, swIAV isolation was more successful on MDCK cells (23/32) compared to ECE (8/32) and nasal swabs were more suitable for virus isolation than oral fluid.

### Influence of conservation time and temperature on swine influenza A virus infectivity and qRT-PCR detectability in porcine oral fluid

**Fig 2:**
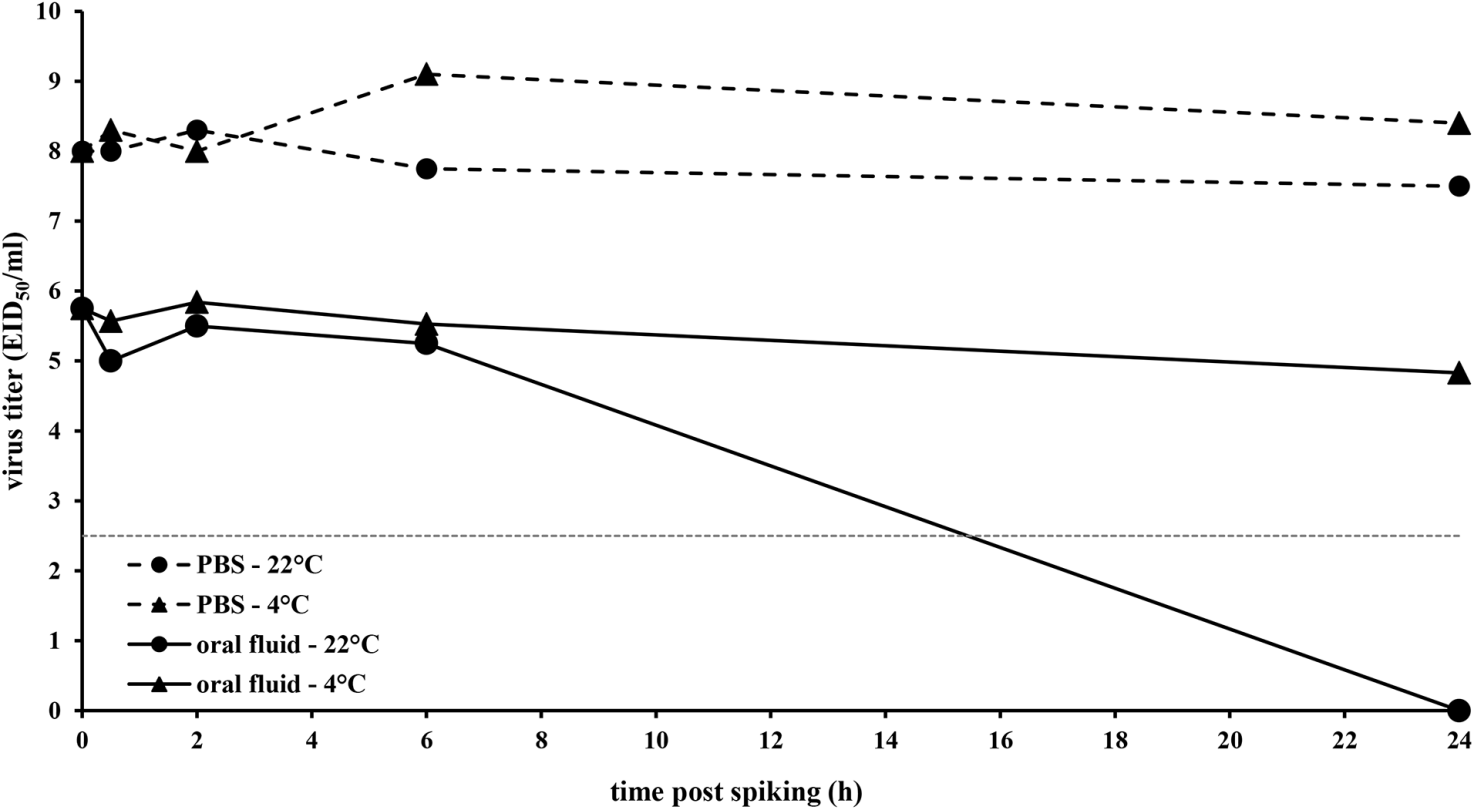
Influence of conservation time and temperature on swine influenza A virus infectivity in porcine oral fluid as determined by virus titration on embryonated chicken eggs. H1N1 virus stock was spiked in porcine oral fluid or PBS to a final concentration of 1 x 10^8.7^ EID50 / mL and conserved either at 4°C or at room temperature (22°C ± 2°C). At 0, 0.5, 2, 6 and 24h after spiking, aliquots of 2 mL were taken and incubated with PBS supplemented with antibiotics (9:1) for 1h at room temperature. Afterwards 10-fold dilutions were made and per dilution, 5 eggs were inoculated with 100μl each. Titers were calculated using the method of Reed and Muench.

Control experiments whereby swIAV was spiked in PBS to an initial virus concentration of 1 x 10^8.7^ EID_50_ / mL showed that swIAV was stable in PBS during 24 h (Fig 2). No obvious loss in virus infectivity as measured by virus titration in ECE occurred during this time period, neither when conserved at 4°C (titer 24 h after spiking: 1 x 10^8.4^ EID_50_ / mL), nor at room temperature (titer 24 h after spiking: 1 x 10^7.5^ EID_50_ / mL). In contrast, spiking the same amount of swIAV in oral fluid immediately resulted in a thousand fold reduction in virus titer (titer of 1 x 10^5.75^ EID_50_ / mL at 0 h post spiking). When the spiked oral fluid was further conserved at 4°C, the remaining amount of infectious virus seemed to remain stable during a 24 h period since viral titers of 1 x 10^5.84^, 1 x 10^5.53^ and 1 x 10^4.83^ EID_50_ / mL were found at 2, 6 and 24 h post spiking, respectively. Incubation of swIAV in oral fluid at room temperature did not result in a further reduction of virus infectivity during the first 6 h post spiking (1 x 10^5.25^ EID_50_ / mL). Thereafter, however, swIAV infectivity further decreased and dropped below the limit of detection (1 x 10^2.5^ EID_50_ / mL) at 24 h post spiking.

**Fig 3:**
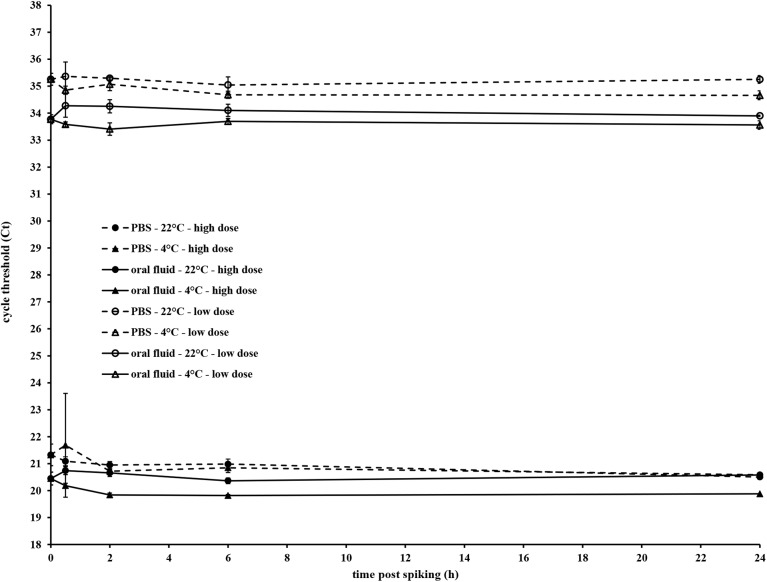
Influence of conservation time and temperature on qRT-PCR detectability of swine influenza A virus in porcine oral fluid. H1N1 virus stock was spiked in porcine oral fluid or PBS to final concentrations of either 1 x 10^8.7^ EID_50_ / mL or 1 x 10^4.7^ EID_50_ / mL. The spiked samples were conserved either at 4°C or at room temperature (22°C ± 2°C) and at 0, 0.5, 2, 6 and 24 h after spiking, aliquots were collected and stored at -80°C. After RNA extraction using the MagMAX Pathogen RNA/DNA kit (Life Technologies), swIAV RNA was amplified with the Vetmax Gold swIAV detection kit (Life Technologies). Mean Ct values (± standard error) of three independent replicates are shown.

qRT-PCR analysis of oral fluid samples that were spiked with swIAV to concentrations of 1 x 10^8.7^ and 1 x 10^4.7^ EID_50_ / mL showed that the observed drop in infectivity after spiking of swIAV in oral fluid did not coincide with considerable changes in the detectability of viral nucleic acids (Fig 3). The Ct values obtained immediately after spiking of swIAV in PBS and oral fluid were slightly higher in PBS than in oral fluid (indicating a higher retrieved number of viral nucleic acids from oral fluid than from PBS) for both the high (21.3 vs 20.5) and low (35.3 vs 33.6) spiked dose. It can therefore be concluded that spiking of swIAV in oral fluid did not coincide with an immediate reduction of viral nucleic acids, as was observed for the virus infectivity. Also further conservation of the spiked oral fluid samples did not result in important changes in the detectable amount of swIAV nucleic acids. The mean Ct values obtained at the different time points till 24 h after spiking did not differ more than 0.5 Ct, irrespective whether the samples were conserved at 4°C or at room temperature and this for both the high and low starting concentration. Similar results were obtained in the control experiments whereby swIAV was spiked in PBS.

## Discussion

Collection of a sufficient amount of oral fluid is the first requisite to be able to progress to downstream diagnostics. Prickett et al. [30] described a method to efficiently collect pen-based oral fluid samples from pigs, whereby ropes are hung in a pen. The exploratory behaviour of pigs makes them chew on the rope and moisten it with oral fluid that can easily be collected afterwards. The oral fluid collections in this study showed however to be more difficult and dependent on individual animal behaviour. As recently described for a similar study [33], the observed low success rate of oral fluid collection was probably related to the young age of the pigs used and to their individual housing conditions. This is an important aspect to take into consideration when planning future experiments with individually housed piglets.

The laboratory diagnosis of influenza virus infection has typically relied upon the detection of the virus in nasal swabs. Serology to detect antibodies is of low value for swIAV surveillance because vaccination against swIAV is based on inactivated H1N1 and H3N2 vaccines and current serologic tests do not differentiate between vaccinated and infected animals [34]. Only in holdings where no swIAV vaccination is practiced, testing of paired sera (acute and convalescent serum) might be useful. Therefore, virological assays are currently preferred over serology for surveillance. To allow virus detection, nasal swabs need to be collected during the acute phase of infection which is limited in time, mostly between 1 to 4–5 dpi [27]. The results of this study are in agreement with these findings since for both the H1N1 and H3N2 strain used, virus could be detected in nasal swabs from 1 till 5 dpi.

Recent publications have suggested the utility of pen-based oral fluid samples for the virological diagnosis of swIAV by qRT-PCR [28–29,35]. It was described that the probability to detect swIAV in oral fluids and nasal swabs by qRT-PCR was equivalent till 6 dpi and then higher in pen-based oral fluid samples till the end of the experiment at 16 dpi. This is in agreement with our results showing that in experimentally infected and individually penned animals swIAV could be detected by qRT-PCR for a longer time period and with a higher detection rate in porcine oral fluid compared to nasal swabs. Especially noteworthy was the detection of swIAV RNA till at least 21 dpi in 25% of the oral fluid samples, while all nasal swabs already became negative at 7 dpi.

The virus detected in nasal swabs most probably originates from local virus replication, which normally occurs up to 5 days after intranasal inoculation. H1N1 swIAV is only detectable by virus isolation till 6 dpi in nasopharynx, tonsils, trachea, and some lung parts [36]. The reported presence of only limited numbers of single swIAV positive cells in nasal mucosa and nasopharynx after H1N1 swIAV infection in pigs [36] can help to explain the fast drop in qRT-PCR positive nasal swabs after local virus replication has stopped. The influenza RNA detected up till 21 dpi in oral fluids does probably not originate from such local virus replication in the nasal mucosa, but might rather be explained by the detection of viral RNA in expectorated sputum. Such sputum contains cellular debris from the lower respiratory tract (e.g. trachea, bronchi, aveoli, bronchiole) [37] which is known, just like the interstitial and alveolar macrophages, to contain high amounts of viral RNA and antigen in naturally infected pigs [36, 38]. The long turnover time of these cells that are potential sources of viral RNA in expectorated sputum—one to three weeks for epithelial cells in trachea, large bronchi and small bronchi in adult mice and 35 days for the alveolar macrophages [39, 40]–seems in line with the prolonged swIAV detection in oral fluid. Also our finding that the level of viral nucleic acids remained unaltered during a 24 h incubation period in oral fluid helps to the explain why swIAV can be detected for a prolonged period in oral fluid.

The possibility to detect the H3N2 strain in both nasal swabs and oral fluid by qRT-PCR after a previous infection with a H1N1 strain was another important observation. This might be explained by the fact that although cross-protective immunity has been described for swIAV [41], heterosubtypic immunity induced by natural infection is mostly weak [42]. As a result, intranasal inoculation with H1N1 induces only partial protection against subsequent infection with other influenza A virus subtypes, like the H3N2 strain.

Besides the importance to detect swIAV RNA in diagnostic samples for surveillance purposes, it might also be important to isolate the virus from the diagnostic sample for further characterization. Our results show that swIAV isolation was more successful on MDCK cells than on ECE. These findings suggest that although ECE are still considered the golden standard for isolation of swIAV from infected animals [43], MDCK cells might be superior to ECE for the detection of certain subtypes/strains of swIAV. In this context, it has already been reported that the success rate of one of both isolation systems is mainly dependent upon the virus subtype [44]. Furthermore, our results indicate that swIAV isolation is more efficient from nasal swab samples than from oral fluid samples, even if they are collected from the same pig at the same time point. Similar observations have been reported before [28,29] and suggest that oral fluid has a negative impact on swIAV infectivity. This is supported by our laboratory experiments, showing that spiking of swIAV in oral fluid results in an immediate thousand fold reduction in virus titer, thereby reducing the chance on a positive virus isolation from an oral fluid sample during diagnosis. Furthermore, continued conservation of the spiked oral fluid samples at room temperature further decreased the virus infectivity till it was completely lost after 24 h. This rapid loss of infectivity in oral fluid seems to be in line with the observation that swIAV was only successfully isolated from oral fluid samples collected at 1 day post infection with the H1N1 strain. With regard to diagnosis, these results indicate that freshly collected oral fluid samples should be tested in the briefest delay to maximize virus detection and strongly argue to cool oral fluid samples as fast as possible upon collection and conserve them at 4°C when virus isolation is envisioned.

The causative factor for the reduction of swIAV infectivity in oral fluid has not yet been irrefutably identified. Goodell et al. [29] suggested that the lower isolation rates from oral fluid samples compared to nasal swab samples might be caused by the presence of anti-influenza antibodies in the oral fluid samples. This seems to be in agreement with our incapability to isolate swIAV from oral fluid collected after the H3N2 infection performed 21 days after the primo infection with H1N1. It should however be kept in mind that only three oral fluid samples with Ct < 30 were available for virus isolation. Detmer et al. [28] suggested that glycoprotein–340 and MUC5B which are present in human saliva and have inhibiting and neutralizing activities against influenza virus strains might contribute to these lower detection ratios. Furthermore also other endogenous mucosal antiviral factors present in oral fluid [45] or physicochemical properties of oral fluid like its hypo-osmolar nature [46–48] might equally be responsible for the antiviral properties. Our results showing that qRT-PCR detectability of swIAV remains unaltered during a 24 h incubation period in oral fluid indicate that the reduced infectivity is probably not the result of viral genome degradation by RNases known to be present in oral fluid [49] and that the importance of the components mentioned above should be studied further. Another explanation for the incapability to isolate swIAV from the qRT-PCR positive oral fluid samples collected during our in vivo experiment that cannot be excluded for the moment is the possibility that simply no infectious virus was present in those samples and that the positive qRT-PCR results originate from viral RNA in expectorated sputum as discussed in a previous paragraph.

Finally, it should be emphasized that oral fluid was collected with polyester ropes in this study while most studies rely on cotton ropes to collect oral fluid from pigs. Although it has been shown that rope material influences downstream antibody and hormone detection [50–52], multiple studies have shown that polyester is a suitable material to collect samples for downstream virus detection by PCR or isolation. Decorte et al. [33] showed that there was no difference in the detection of PRRSV virus by PCR when oral fluid was collected from pigs either with cotton or polyester ropes. Other studies showed that polyester swabs were suitable for swIAV sample collection from pigs for downstream PCR detection [53] and that polyester swabs were equally suitable as cotton swabs for downstream pseudorabies virus and bovine herpes virus 1 isolation [54–55]. Furthermore, the obtained results that swIAV could be detected both in nasal swabs and polyester rope collected oral fluid from all pigs from the same time point (1 dpi) confirm the suitability of polyester ropes for swIAV sample collection.

Although further research is advisory to evaluate the influence of virus strain or subtype and inoculation dose, our data indicate that porcine oral fluid samples collected with ropes hold potential for diagnostic purposes seen the possibility to detect swIAV RNA for a longer period than in nasal swabs. The difficulty to isolate swIAV from these oral fluid samples could however pose a drawback and has to be studied more intensively.

## Supporting Information

S1 Table Cycle threshold (Ct) values obtained for the detection of swine influenza A virus. The Vetmax Gold swIAV kit (Life Technologies) was used for the detection of swine influenza A virus RNA by qRT-PCR in nasal swab samples (A) and oral fluid samples (B) of pigs sequentially infected with swine influenza A strains sw/Gent/28/10 (H1N1) at day 0 and sw/Gent/172/08 (H3N2) at day 21.
(DOCX)
