# Supplementary material for: Detection and Isolation of Swine Influenza A Virus in Spiked Oral Fluid and Samples from Individually Housed, Experimentally Infected Pigs: Potential Role of Porcine Oral Fluid in Active Influenza A Virus Surveillance in Swine
Source: PLoS One. 2015 Oct 2;10(10):e0139586. doi: 10.1371/journal.pone.0139586 (PMC4592207; doi:10.1371/journal.pone.0139586)
Supplement: S1 Table — The Vetmax Gold swIAV kit (Life Technologies) was used for the detection of swine influenza A virus RNA by qRT-PCR in nasal swab samples (A) and oral fluid samples (B) of pigs sequentially infected with swine influenza A strains sw/Gent/28/10 (H1N1) at day 0 and sw/Gent/172/08 (H3N2) at day 21. (DOCX) [file pone.0139586.s001.docx]

**S1 Table.** Cycle threshold (Ct) values obtained with the Vetmax Gold swIAV kit (Life Technologies) for the detection of swine influenza A virus RNA by qRT-PCR in nasal swab samples (A) and oral fluid samples (B) of pigs sequentially infected with swine influenza A strains sw/Gent/28/10 (H1N1) at day 0 and sw/Gent/172/08 (H3N2) at day 21.

| **A** |  |  |  |  |  |  | | |  | |  | |  | |  | |  |  |  |  |
| --- | --- | --- | --- | --- | --- | --- | --- | --- | --- | --- | --- | --- | --- | --- | --- | --- | --- | --- | --- | --- |
| **Virus strain** | **H1N1 (dpi)** | **0** | **1** | **2** | **3** | | **5** | **7** | | **10** | | **14** | | **21** | | **22** | | **23** | **24** | **26** |
|  | **H3N2 (dpi)** |  |  |  |  |  | | |  | |  | |  | | **0** | | **1** | **2** | **3** | **5** |
| **Animal** | **1** | - | 31.9 | 30.2 | 24.6 | - | | | - | | - | | - | | - | | 27.6 | 28.7 | 27.6 | - |
|  | **2** | - | 36.8 | 32.7 | 34.9 | - | | | - | | - | | - | | - | | 30.2 | 33.5 | 34.8 | - |
|  | **3** | - | 30.7 | 29.5 | 29.7 | - | | | - | | - | | - | | - | | - | 33.0 | 33.8 | - |
|  | **4** | - | 26.5 | 29.7 | 27.9 | 36.6 | | | - | | - | | - | | - | | 31.0 | 29.3 | 31.3 | 37.5 |
|  | **5** | - | 35.5 | 31.4 | 32.6 | - | | | - | | - | | - | | - | | - | - | - | - |
|  | **6** | - | 29.0 | 28.2 | 28.0 | - | | | - | | - | | - | | - | | 36.2 | 30.0 | 34.1 | - |
|  | **7** | - | 29.7 | 31.2 | 33.0 | - | | | - | | - | | - | | - | | 34.1 | 29.5 | 31.0 | 36.7 |
|  | **8** | - | 31.9 | 29.8 | 29.6 | - | | | - | | - | | - | | - | | 36.7 | 34.5 | 34.2 | - |
|  | **9** | - | 29.7 | 29.2 | 28.8 | - | | | - | | - | | - | | - | | 34.1 | - | - | - |
|  | **10** | - | 32.7 | 30.7 | 31.4 | - | | | - | | - | | - | | - | | 33.8 | 33.4 | 33.2 | - |

| **B** |  |  |  |  |  |  |  |  |  |  |  |  |  |  |
| --- | --- | --- | --- | --- | --- | --- | --- | --- | --- | --- | --- | --- | --- | --- |
| **Virus strain** | **H1N1 (dpi)** | **0** | **1** | **2** | **3** | **5** | **7** | **10** | **14** | **21** | **22** | **23** | **24** | **26** |
|  | **H3N2 (dpi)** |  |  |  |  |  |  |  |  | **0** | **1** | **2** | **3** | **5** |
| **Animal** | **1** | ns | 27.5 | 26.7 | ns | 34.0 | 34.7 | ns | 34.9 | 35.7 | ns | ns | ns | ns |
|  | **2** | ns | 36.5 | 36.1 | ns | - | ns | - | ns | - | 29.9 | 30.0 | ns | ns |
|  | **3** | - | 27.7 | 26.4 | ns | ns | 33.1 | ns | 34.6 | 37.0 | ns | ns | ns | ns |
|  | **4** | ns | ns | ns | ns | ns | ns | ns | ns | ns | 30.4 | 26.5 | 27.6 | 31.9 |
|  | **5** | ns | ns | 31.2 | ns | 33.1 | ns | 36.3 | - | - | - | 36.0 | ns | ns |
|  | **6** | - | 29.4 | 27.6 | ns | 28.3 | 34.9 | ns | ns | - | 32.6 | 30.8 | ns | 33.9 |
|  | **7** | - | ns | ns | ns | ns | ns | - | - | - | 33.8 | ns | ns | 35.3 |
|  | **8** | ns | ns | ns | ns | ns | ns | ns | - | ns | 34.2 | ns | ns | ns |
|  | **9** | - | 27.7 | 30.2 | 27.2 | 34.3 | 34.8 | 36.0 | 36.1 | - | + | 36.0 | 32.3 | - |
|  | **10** | ns | ns | ns | ns | ns | 35.8 | ns | - | - | ns | 31.6 | 29.6 | 35.2 |

ns: No sample available; -: PCR neg
